# Automation of large scale transient protein expression in mammalian cells

**DOI:** 10.1016/j.jsb.2011.04.017

**Published:** 2011-08

**Authors:** Yuguang Zhao, Benjamin Bishop, Jordan E. Clay, Weixian Lu, Margaret Jones, Susan Daenke, Christian Siebold, David I. Stuart, E. Yvonne Jones, A. Radu Aricescu

**Affiliations:** Division of Structural Biology (STRUBI), Wellcome Trust Centre for Human Genetics, University of Oxford, Roosevelt Drive, Oxford OX3 7BN, UK

**Keywords:** Automated tissue culture, Eukaryotic expression system, HEK 293 cells, Transient transfection, HYPERFlask

## Abstract

Traditional mammalian expression systems rely on the time-consuming generation of stable cell lines; this is difficult to accommodate within a modern structural biology pipeline. Transient transfections are a fast, cost-effective solution, but require skilled cell culture scientists, making man-power a limiting factor in a setting where numerous samples are processed in parallel. Here we report a strategy employing a customised CompacT SelecT cell culture robot allowing the large-scale expression of multiple protein constructs in a transient format. Successful protocols have been designed for automated transient transfection of human embryonic kidney (HEK) 293T and 293S GnTI^−^ cells in various flask formats. Protein yields obtained by this method were similar to those produced manually, with the added benefit of reproducibility, regardless of user. Automation of cell maintenance and transient transfection allows the expression of high quality recombinant protein in a completely sterile environment with limited support from a cell culture scientist. The reduction in human input has the added benefit of enabling continuous cell maintenance and protein production, features of particular importance to structural biology laboratories, which typically use large quantities of pure recombinant proteins, and often require rapid characterisation of a series of modified constructs. This automated method for large scale transient transfection is now offered as a Europe-wide service via the P-cube initiative.

## 1 Introduction

Fewer than 3% of the protein structures currently deposited in the Protein Data Bank (PDB) were solved using samples produced in mammalian cells (Nettleship et al., 2010), despite the recent emergence of rapid and cost-effective transient expression methods (Aricescu et al., 2006; Durocher et al., 2002; Meissner et al., 2001). However, the problems encountered for the large-scale expression of many proteins of eukaryotic, especially human, origin have increasingly focused attention on mammalian cell-based expression systems as a route for the production of targets that prove challenging in heterologous prokaryotic and insect cell expression hosts. In mammalian expression systems the protein, or complex, can be expressed in its native cell type, under physiological conditions, with numerous molecular systems working together for efficient production and quality control. Unfortunately, the large-scale expression of eukaryotic proteins in a stable and soluble form is still a major bottleneck. This is due, in part, to the time consuming process required to maintain and expand cells, and to perform transient transfections. Automated mammalian cell culture using stably transfected cell lines is becoming increasingly prevalent in many stages of drug discovery (Szymanski et al., 2008). In its current form however, time considerations mean that it is of less use in modern structural biology laboratories, where many different constructs are typically processed in parallel and rapid feedback loops, supported by automated protein crystallisation (Walter et al., 2003, 2005), may necessitate construct re-design within a two week cycle (Aricescu et al., 2006). Manual cell culture, typically performed by experienced staff, is highly repetitive and requires considerable time commitment and is therefore an ideal candidate for automation. Automated tissue culture using the SelecT robot has already been reported for stable cell lines used in drug discovery programmes, demonstrating advantages such as consistency between operators and batches, ease of use and reduced risks of contamination (Szymanski et al., 2008). Robotic transient transfection poses a significant challenge for such existing cell culture systems however, largely due to the requirement to exchange media and deliver various transfection mixtures to flasks in a linear sequence within a strict time frame.

We present here simple and reproducible protocols for the automated large-scale transient transfection of constructs cloned into the pHLsec vector (Aricescu et al., 2006) in two human cell lines: HEK 293T and the N-glycosylation deficient HEK 293S GnTI^−^ (Reeves et al., 2002). A survey of 75 different protein batches, expressed using this robot over a four month period, allowed us to examine the distribution of pure protein yields and assess the usefulness of such a machine in the demanding environment of an academic X-ray crystallography laboratory.

## 2 Materials and methods

### 2.1 Vectors and cell lines

The vector chosen for development of an automated transfection protocol was pHLsec. This vector was built on the pLEXm backbone (Aricescu et al., 2006) and contains: pBR322 origin of replication giving the high copy number in *Escherichia coli* needed to obtain the high amounts of plasmid DNA required for transient transfection; ampicillin resistance; a cytomegalovirus enhancer; chick β-actin promotor to give high levels of expression; the rabbit β-globin intron to increase RNA production; and a poly-A signal to increase RNA stability. Two cell lines were chosen for the development of automated transient transfection protocols for glycosylated mammalian proteins: HEK 293T (ATCC CRL-11268), grown in the presence of the N-glycosylation inhibitor, kifunensine (Chang et al., 2007) added immediately post-transfection at 1 mg/L final concentration, and the N-acetylglucosaminyltransferase I-negative HEK 293S GnTI^−^ cells (Reeves et al., 2002), which are unable to synthesize complex glycans. These cell lines were chosen due to ease of handling, robust growth rates, excellent transfection efficiency, high capacity for recombinant protein expression and low cost media requirements. Both cell lines are also routinely used for manual transient transfections, thus allowing a direct comparison between automated and manual protocols.

### 2.2 DNA purification

The Endotoxin-Free Plasmid High Speed Giga Kit (Qiagen) was used to purify plasmid DNA. High-quality DNA is essential for successful transfections and only samples with an OD_260/280_ ratio of 1.8 or higher are suitable. The pLEXm-derived plasmids are very high copy number, therefore one can expect between 12 and 15 mg of pure DNA from a 2 L overnight bacterial culture (*E. coli* DH5α strain). It is essential that DNA samples are sterile, so care was taken to wash the DNA precipitates with 70% ethanol before dissolving them in sterile 10 mM Tris pH 8.

### 2.3 Transfection reagent

Polyethylenimine (PEI) is an affordable and highly efficient transfection reagent. The PEI used in both manual and automated protocols is ‘25 kDa branched’ (Sigma–Aldrich), which was reported to be effective in transfecting various HEK293 cell lines (Aricescu et al., 2006; Durocher et al., 2002). Stock solutions were made in water at 100 mg ml^−1^, the pH adjusted to 7 with HCl, filter sterilised and aliquoted. Aliquots and working solutions of 1 mg ml^−1^ can be stored frozen at −20 °C for long periods of time (months) without a reduction in transfection efficiency.

### 2.4 Cell maintenance protocol

Cells were grown in Dulbecco’s Modified Eagle’s Medium (DMEM high glucose, Sigma) supplemented with l-glutamine, non-essential amino-acids (Invitrogen) and 10% Foetal Bovine Serum (FBS, Invitrogen). Cells were manually cultured from stocks stored at −196 °C, using a T25 flask (Corning). On reaching 90% confluence, cells were detached using trypsin–EDTA (Sigma), seeded in a T75 flask (Corning or Greiner) and finally transferred to a barcoded T175 flask (Corning or BD-Falcon) and imported into the CompacT SelecT Cellbase robot (The Automation Partnership, Royston, UK), where all subsequent cell maintenance was automated.

### 2.5 Automated cell maintenance and seeding

Cells were harvested from 90% confluent T175 flasks, analysed by the Cedex automated cell counter and diluted to a concentration of 1.6 × 10^6^ cells ml^−1^ in DMEM, 10% FBS. New bar-coded T175 flasks were then called from the CompacT SelecT Cellbase incubator hotel and seeded by pipetting 10^7^ cells per flask, supplemented with DMEM, 10% FBS to a final volume of 30 ml. The robotic Stäubli arm then swirled the flask to mix the cells before placing it in the hotel incubator. Cells were passaged twice a week, using a trypsin–EDTA mix (Sigma) for detachment, to a maximum of 20 passages before new aliquots were retrieved from liquid nitrogen storage. Triple flasks (Nunc) were seeded with 2 × 10^7^ cells, topped up to 110 ml with DMEM/10% FBS. Ten layer HYPERFlasks (High Yield PERformance Flask, Corning) were seeded with 6 × 10^7^ cells, and topped up to 555 ml with DMEM, 10% FBS. Both flask types were returned to the robot incubator for four days, irrespective of the cell line used, prior to transfection. We provide the protocol codes for these procedures in Supplementary Figs. 1–3.

### 2.6 Automated transient transfection protocol

The transfection cocktail was prepared manually prior to the automated transient transfection of Triple flasks or HYPERFlasks. To transfect 12 Triple flasks, 1.8 mg plasmid DNA was chloroform extracted (to ensure sample sterility) prior to addition of 360 ml serum free DMEM. Mixing was followed by the addition of 3.6 ml PEI stock (1 mg ml^−1^) with brief swirling followed by a 10 min incubation at room temperature to allow DNA-PEI complex formation. The robot can follow only linear protocols, as it has but one arm. The automated protocol for Triple flasks differed from that used manually as the robot cannot work quickly enough to transfect 12 Triple flasks (a typical batch size, providing 1.2 L of conditioned media) in less than the hour before transfection efficiency begins to drop (see Section 3.1). To prevent this problem, it was necessary to stack two programs: the first program adds the transfection cocktail to each of the 12 flasks (30 ml per Triple flask), and when this procedure is completed for all the flasks, a second program is run to top up the flasks to the final volume of 100 ml with DMEM, containing a final FBS concentration of 1.6% (Supplementary Fig. 4). To transfect one HYPERFlask, chloroform extracted DNA was added manually to 100 ml serum-free DMEM, mixed, supplemented with 2 ml PEI stock (1 mg ml^−1^) and briefly mixed by swirling followed by a 10 min incubation at room temperature to allow DNA-PEI complex formation. Afterwards, the automated transfection protocol was started. Flasks seeded four days prior to transfection were taken from the incubator and media removed by pouring into the waste receptacle. The DNA:PEI complexes were then added to the flasks (100 ml per HYPERFlask), followed by low serum DMEM (455 ml per HYPERFlask), resulting in a final FBS concentration of 1.6%.

### 2.7 Manual transfection in roller bottles

This was typically performed as described in Aricescu et al. (2006). Half a milligram of plasmid DNA was used for transfection in each 2125 cm^2^ expanded surface roller bottle (Greiner). Briefly, the DNA solution was added to 50 ml serum-free DMEM, mixed, supplemented with 1 ml PEI stock (1 mg ml^−1^) and vortexed for 10 s. This solution was incubated for 10 min at room temperature to allow DNA–PEI complex formation. During complex formation, media from the roller bottles was changed, lowering the serum concentration (200 ml of DMEM, 2% FBS, were added per roller bottle). Finally, the DNA–PEI complex was added to each bottle, briefly rotated to allow mixing, after which the cells were placed in the incubator. Approximately 4–7 days later, conditioned medium was ready for collection and protein purification.

### 2.8 Fluorescence microscopy

Imaging of transfection efficiency using EGFP-pLEXm constructs was carried out with a Nikon Eclipse TE2000U fluorescence inverted microscope equipped with a Hamamatsu C4742-95 Orca low-noise B&W CCD and using IPLab imaging software to capture and process images.

### 2.9 Western blot analysis

Small aliquots of conditioned media (10 μl) were analysed by Western blotting using the PentaHis monoclonal antibody (1:1000 dilution, Qiagen) and goat anti-mouse IgG peroxidase-conjugated secondary antibody (diluted 1:2000, Sigma). The signal was visualised by chemiluminescence using the ECL kit and hyperfilm (GE Healthcare).

### 2.10 Shh protein purification

For the comparison between large-scale automated and manual transfection procedures secreted His-tagged constructs of the N-terminal domain of mouse sonic hedgehog (Shh) were purified using an immobilised metal affinity chromatography (IMAC) batch procedure. Conditioned media were filtered through a 0.2 μm membrane (Express filter, Millipore) and dialysed against 25 mM phosphate buffer saline (PBS, pH8.0) at 4 °C to prevent metal stripping from the affinity chromatography column. IMAC purification was performed using cobalt-coated Talon beads (Clontech) which were washed with 7.5 mM imidazole in PBS (pH 8.0), and proteins eluted by addition of 250 mM Imidazole in 10 mM Tris, pH 8.0, 500 mM NaCl. The proteins were further purified by size exclusion chromatography on a Superdex200 10/30 (GE Healthcare) column.

## 3 Results

### 3.1 Automated transient transfection: protocol design, implementation, optimisation

**Fig.1:**
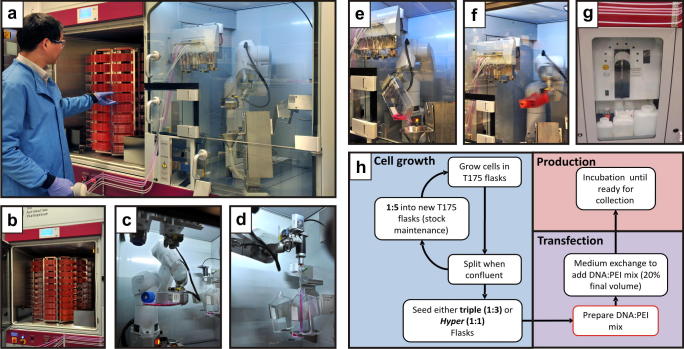
The different CompacT Cellbase robot modules, employed for cell maintenance, seeding and transfection. (a) The two robot units: incubator/hotel on the left, accessible to the operator; laminar flow cabinet for flask processing, on the right. The robot arm can access the cell flasks within the incubator carousel through an automated internal door which is synchronised with carousel turning. All liquid cell culture reagents, such as media, PBS and trypsin are connected to the system through silicon tubing and driven via peristaltic pumps. There are 10 pumps for up to 10 lines of different cell culture liquids. The robot system also coordinates the capping or decapping of flasks and presents the flask to the relevant media line, delivering precise amounts of liquid. The system uses 10 ml pipettes for transferring cell suspensions or adding solutions from the static liquid holder. (b) Carousel consisting of nine vertical hotels, each with a ten flask capacity, for cell maintenance and four hotels with ten positions for new flasks. (c) The Stäubli robot arm gripping a single flask, ready to be returned to the carousel incubator. (d) Splitting flasks using the pipette head to transfer cells. (e) Pouring media from a flask into the waste receptacle. (f) The robot arm performing the “hyperswirl” command after transfection; the “cocktail bar” can also be seen on the left of the photo. (g) The Cedex cell-counting module. (h) Workflow of automated cell culture, including cell maintenance, transfection and protein production steps. The only step performed manually is the DNA:PEI mix preparation.

The CompacT SelecT robot (Fig. 1a–g), recently developed by The Automation Partnership (Royston, Cambridge, UK), is a smaller version of the SelecT automated cell culture platform. This system retains more than 70% of the SelecT capacity and, importantly, is able to manipulate 10 layer HYPERFlasks. Typically it is used for cell-based drug screening or protein production using stable cell lines (Szymanski et al., 2008). The CompacT Cellbase, a CompacT SelecT without a plating module, was chosen for our mammalian protein expression. The system consists of two compartments. The first is a humidified 5% CO_2_, 37 °C carousel incubator/flask hotel unit (Fig. 1b) with space for 40 new input flasks and 90 production flasks for cell maintenance and transfection. The second is a laminar flow compartment containing a Stäubli robotic arm (Stäubli Robotics, Faverges, France) (Fig. 1c), a pipette head (Fig. 1d), a waste receptacle and “cocktail bar” (Fig. 1e). The laminar flow compartment also has a barcode reader to track flasks and a flask de-capper (Fig. 1f). The unit also incorporates a Cedex automated cell counting module (Fig. 1g). Cells are automatically maintained by regular harvesting and seeding of T175 flasks, while production is performed in Triple flasks and HYPERFlasks, following the flowchart described in Fig. 1h.

**Fig.2:**
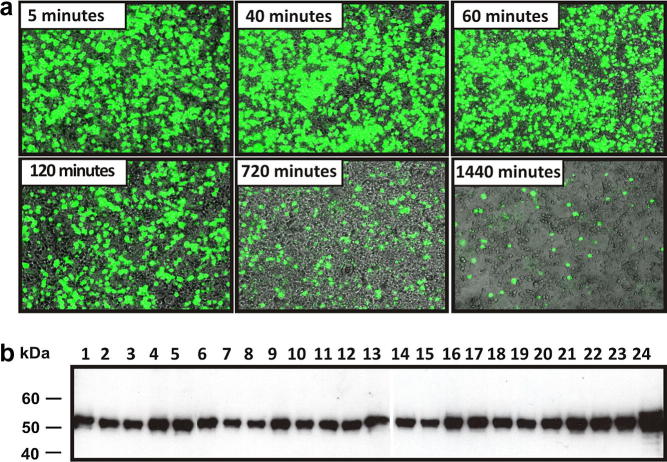
Optimisation of the transient transfection protocol and reliability tests. (a) Cells transfected by the robot at different time points after PEI:DNA mixing. Expression of EGFP allowed visualisation of transfection efficiency following different incubation periods. Transfection protocols were designed to cater for this decrease in efficiency over time. (b) Western blot analysis of samples from 24 separate T175 flasks after automated transfection with the pHLsec vector encoding the same 50 kDa glycoprotein construct.

We designed and extensively tested a series of protocols for routine cell maintenance as well as transfection in various flask formats (protocol codes and schematic diagrams are shown in Supplementary online materials). To establish the optimal transfection protocol for Triple flasks and HYPERFlasks, a pLEXm vector encoding the EGFP was used to allow visualisation and quantification of cell transfection efficiency. A series of time points after addition of PEI to DNA was investigated, in order to determine the maximum number of efficient transfections achievable with one batch of DNA:PEI mix. Optimal transfection was achieved when adherent cells reached 90% confluence. DNA requirements were 150 μg per Triple flask and 1 mg per HYPERFlask. To determine the optimal number of flasks which could be efficiently transfected with one batch of DNA:PEI mix, a range of incubation times was investigated. Transfection efficiency, determined by monitoring live-cell EGFP fluorescence, remained stable for one hour after mixing PEI with DNA, but subsequently started to decrease (Fig. 2a). Each HYPERFlask takes eight minutes to complete the transfection protocol, therefore no more than six HYPERFlasks can be transfected from one batch of DNA-PEI mixture if efficiency is to be maintained.

The reliability of the whole procedure was tested by using this system to seed and independently transfect (during the same day) 24 different T175 flasks with the same pHLsec construct, encoding a 50 kDa glycoprotein. A sample of media from each flask was collected and analysed by western blot with an anti-His tag antibody, showing consistent protein expression (Fig. 2b) when compared to the two orders of magnitude variation which, in our experience, can arise from human error.

### 3.2 Comparison between large-scale automated and manual transfection procedures

**Fig.3:**
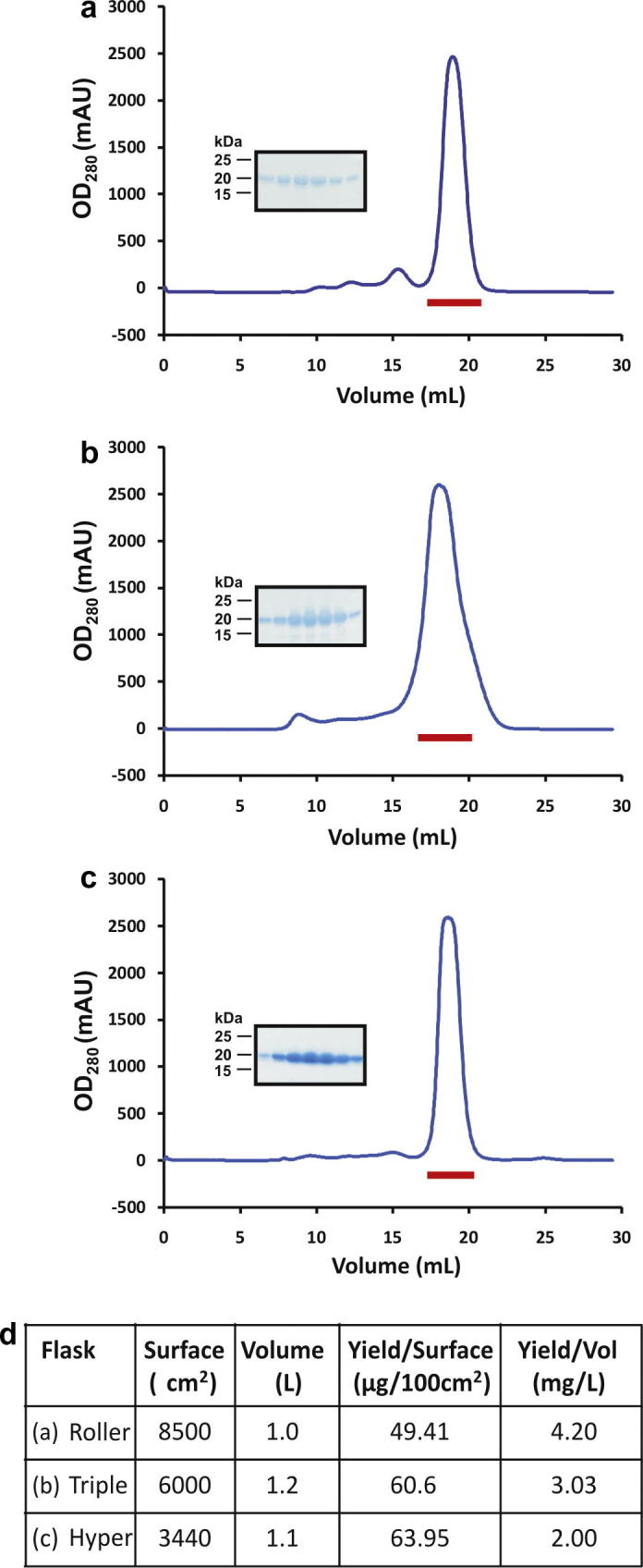
Comparison of three protein expression strategies. The secreted form of the mouse Shh N-terminal domain was purified from conditioned media by IMAC and SEC (blue traces) and analysed by SDS–PAGE (inset) of peak fractions (shown by a red bar on SEC traces). The protein was produced using: (a) four Roller bottles seeded and transfected manually, (b) 12 Triple flasks seeded and transfected by the robot and (c) two HYPERFlasks, seeded and transfected by the robot. (d) Table summarising the experimental output of the above experiment.

It was important to ensure that the efficiency of protein production resulting from the automated transfection protocol matched that of the manual system in order to demonstrate that automated transient transfection was a viable alternative. To this end, test expression experiments using the N-terminal signalling domain of mouse sonic hedgehog (Shh), solubly secreted in the conditioned medium (Bishop et al., 2009), were performed in three configurations: four roller bottles manually transfected with 2 mg DNA; automated transfection of 12 Triple flasks with 1.8 mg DNA; and automated transfection of two HYPERFlasks with 2 mg DNA. Four days post transfection, media were harvested and purified before analysing the protein samples by SEC and SDS–PAGE (Fig. 3a–c). The construct was well expressed in all procedures (Fig. 3d), demonstrating that the robot is a viable alternative to manual tissue culture. The smaller footprint of HYPERFlasks provides an advantage over the Triple variant, occupying fewer slots in the incubator hotel for a comparable output. However, the protein produced is at roughly half the concentration of that observed for roller bottles. In terms of real “hands-on” time, the benefit of automation is directly proportional to the scale of the experiment. A typical experiment of manual transfection in 12 roller bottles takes approximately 30 min of operator input, similar to automated transfection of six HYPERFlasks (giving an equivalent volume of media). Doubling the output on the robot adds 10 further minutes of operator time, significantly less than doubling the number of roller bottles handled manually, which typically takes a lot longer due to operator fatigue. The greatest benefit of automation results from cell maintenance and seeding procedures, which require minimal operator input. Media and flasks must be supplied by an operator, but all subsequent procedures can then be fully automated, running overnight if necessary.

### 3.3 Analysis of a 4-month production run

**Fig.4:**
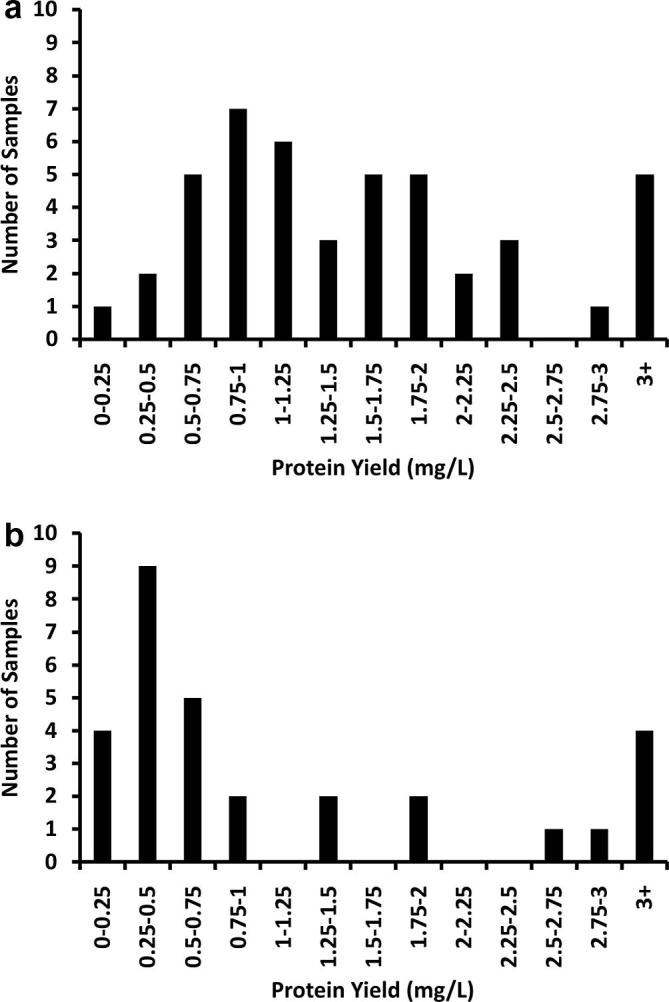
Pure protein yields obtained from HYPERFlasks during a four-month automated production run in STRUBI. (a) 42 samples produced in HEK 293T cells and (b) 33 samples produced in HEK 293S GnTI^−^ cells. Each sample represents an individual construct which was transfected in a minimum of two and maximum of twelve HYPERFlasks.

Data from HYPERFlask transient transfections performed on the CompacT SelecT Cellbase robot over a period of four months, using both HEK 293T and HEK 293S GnTI^−^ cells and using only constructs confirmed to be secreted in small-scale experiments, revealed a broad range of pure, crystallisation grade, protein yields (Fig. 4). It should be noted that constructs showing low expression in small scale tests were often put forward for automated transfection as the yields from manual protocols had been found to be typically more variable. These data represent the efforts of many users with different skill levels, handling 75 different samples of varied molecular weight, number of N-linked glycosylation sites, fold and number of domains and oligomeric state. An overall trend of higher expression levels in HEK 293T (Fig. 4a) versus HEK 293S GnTI^−^ cells (Fig. 4b) was observed, but in all cases the protein quantities obtained were sufficient to perform extensive crystallisation trials using nanolitre scale technologies (Walter et al., 2003, 2005). The inherent abilities of the two cell lines to produce recombinant proteins may be responsible for the observed differences, however other factors such as the addition of kifunensine, an α-mannosidase inhibitor used to reduce the complexity of N-linked glycosylation (Chang et al., 2007), which inhibits ER-associated degradation (Tokunaga et al., 2003) cannot be excluded.

## 4 Discussion

The automated protocols described here are equivalent to their manual counterparts in terms of the quality and quantity of secreted mammalian proteins produced. The initial investment in equipment, in the order of £500,000, and the ∼20% higher consumables cost should be considered in balance with a ∼3-fold increase in output above the physical ability of one experienced tissue culture operator. In addition to reproducibility and robustness of the process, the combined efforts of multiple robot operators can provide a significant benefit in long term. From October 2009 to November 2010, automated transfection protocols were responsible for the production of ∼300 protein batches in our laboratory. Secreted proteins produced ranged in size from 10–200 kDa (Coles et al., 2011; Janssen et al., 2010; Malinauskas et al., in press; Seiradake et al., 2010). Separate to the statistics presented (Fig. 4) we have also succeeded in producing intracellular and membrane proteins using the automated large scale transient expression system, however, our experience for these classes of proteins is as yet more limited. What are the benefits of the automated protocols compared to manual methods? The most obvious is the increased capacity for cell culture and protein production which becomes available, whilst human effort is released for other tasks. The manual system is entirely dependent on the skills and presence of an experienced cell culture scientist. In the automated system, there is no such constraint and the vicissitudes of human error are also reduced. As long as media reservoirs are filled as needed and flasks loaded into the robot, automated cell culture can continue 24 h a day 7 days a week without break. Transient transfections, however, still require the presence of an operator to prepare the cocktail of reagents and thus can only be completed during normal working hours. With efficient time-tabling, all fully automated procedures can be completed out of normal working hours and the machine monitored and operated remotely via an internet based video link and control software if required. A further advantage over the manual roller bottle system is that due to the low volume of media required in the roller bottles, HEK 293T cells in the presence of kifunensine typically survive for only 4 days post transfection while in HYPERFlasks efficient gas exchange, allowed by the permeable membrane on which the cells grow, results in cells producing proteins for up to 10 days post-transfection. For HEK 293S GnTI^−^ efficient expression time is increased from a maximum of 7 days post-transfection in roller bottles, to 14 days post-transfection in HYPERFlasks. This increased production time may result in increased yields, however, this advantage is counterbalanced by the increased risks of protein degradation due to storage at 37 °C and proteolysis, thus in general users allowed the same maximum times for the expression of their protein for HYPERFlasks as they did for roller bottles.

Automation of the manual mammalian expression protocol, based on the pLEXm vectors (Aricescu et al., 2006), required some modification to allow for the limitations of the robot. Flasks of suitable geometry include T175 (for maintenance) and either Triple flasks or HYPERFlasks for transient production. Both offer the same ease of handling for the robot, but the Triple flasks occupy three times more volume in the incubator for the equivalent surface area of cell growth. Both types of flask use a lot more media than the roller bottles used in manual transfections. Roller bottles require 250 ml culture media for a surface area of 2125 cm^2^ while Triple flasks minimally require 100 ml each for a surface area of 500 cm^2^ and HYPERFlasks require 555 ml for a surface area of 1720 cm^2^. This then requires a modification of the amount of DNA and PEI used in the transfection cocktail to ensure the correct concentration of both in larger volumes. For roller bottles 0.5 mg DNA with 1 ml PEI was required per bottle. For Triple flasks, 150 μg DNA is required per flask with 250 μl PEI, while for HYPERFlasks, 1 mg DNA per flask with 2 ml PEI. Transfections are carried out in the automated system using peristaltic pump delivery rather than pipettes, as the robot is only able to handle 10 ml pipettes, not a viable option for large volume transfers required by multi-layered flasks. A major drawback of the HYPERFlasks is that they must have 555 ± 1 ml in order to function properly. This requires daily calibration of the pumps, which is not time consuming (less than 10 min) but cannot currently be performed remotely. The T175 and Triple flasks do not require such rigorous calibration; a weekly procedure offers sufficient accuracy.

Having used the CompacT SelecT Cellbase for more than a year, many opportunities for improvements in both the hardware and software have come to light. In terms of hardware, a more consistent delivery system for media would be useful, which would relax the requirement for daily calibration. It is currently necessary to wash the peristaltic pump lines between transfection constructs in order to prevent cross-contamination, a significant problem when the number of flasks transfected with a given construct is small (one or two). Allowing volumes greater than 40 ml into the static liquid holder, located inside the laminar flow unit, would offer greater flexibility in protocol design. Another issue during full time production is the limited space available in the robot incubator. All transfected flasks are routinely removed to free spaces in the nine incubator hotels (which can hold 90 flasks for production and cell maintenance, plus 40 new input flasks at a time). Without the use of additional incubators space would be an issue and, in addition to downstream processing ability, would be a limiting factor in protein production. The robot is currently used to transfect up to 12 HYPERFlasks a day, and to maintain the cells necessary for continuously operating this production cycle. This is close to the maximum efficiency compatible with one operator working normal hours. Finally, in terms of software, the robot is currently unable to use incubation periods to complete other tasks, for example cell passaging. However, even given the current limitations, the automation of large scale transient protein expression in mammalian cells has already had an immensely positive impact on our ability to tackle structural and function studies of challenging biological systems (for example Janssen et al., 2010; Coles et al., 2011). Alternative solutions for automation of small-scale expression and suspension cell culture are available on the market, but remain to be tested in our laboratory (Nettleship et al., 2010).

## 5 Conclusions

The protocols described here are, to our knowledge, the first implementation of an automated tissue culture system for large scale transient expression of secreted recombinant proteins in mammalian cells in a quantity and of a quality suitable for X-ray crystallography. Although somewhat more expensive than manual systems in terms of consumables and requiring significant initial set-up cost, the time saved and the increased productivity makes up for this initial investment. A major advantage of the automated system is the reproducibility of expression between users and at different times as well as the avoidance of simple human error. Finally, the automated protein production system described here is available to all European users as part of the Protein Production Platform (P-cube) (http://www.p-cube.eu/).

## References

[b0005] Aricescu A.R., Lu W., Jones E.Y. (2006). A time- and cost-efficient system for high-level protein production in mammalian cells. Acta Crystallogr. Sect. D.

[b0010] Bishop B., Aricescu A.R., Harlos K., O’Callaghan C.A., Jones E.Y. (2009). Structural insights into hedgehog ligand sequestration by the human hedgehog-interacting protein HHIP. Nat. Struct. Mol. Biol..

[b0015] Chang V.T., Crispin M., Aricescu A.R., Harvey D.J., Nettleship J.E. (2007). Glycoprotein Structural Genomics: Solving the Glycosylation Problem. Structure.

[b0020] Coles C., Shen Y., Tenney A.P., Siebold C., Sutton G.C. (2011). Proteoglycan-specific molecular switch for RPTPσ clustering and neuronal extension. Science..

[b0025] Durocher, Y., Perret, S., Kamen, A. 2002. High-level and high-throughput recombinant protein production by transient transfection of suspension-growing human 293-EBNA1 cells. Nucleic Acids Res. 30, E9. 10.1093/nar/30.2.e9 PMC99848 11788735

[b0030] Janssen B.J.C., Robinson R.A., Perez-Branguli F., Bell C.H., Mitchell K.J. (2010). Structural basis of semaphorin-plexin signalling. Nature.

[b0035] Malinauskas, T., Aricescu, A.R., Lu, W., Siebold, C., Jones, E.Y. in press. Modular Mechanism of Wnt Signalling Inhibition by Wnt inhibitory factor 1. Nat. Struct. Mol. Biol. 10.1038/nsmb.2081 PMC3430870 21743455

[b0040] Meissner P., Pick H., Kulangara A., Chatellard P., Friedrich K. (2001). Transient gene expression: Recombinant protein production with suspension-adapted HEK293-EBNA cells. Biotechnol. Bioeng..

[b0045] Nettleship J.E., Assenberg R., Diprose J.M., Rahman-Huq N., Owens R.J. (2010). Recent advances in the production of proteins in insect and mammalian cells for structural biology. J. Struct. Biol..

[b0050] Reeves P.J., Callewaert N., Contreras R., Khorana H.G. (2002). Structure and function in rhodopsin: High-level expression of rhodopsin with restricted and homogeneous N-glycosylation by a tetracycline-inducible N-acetylglucosaminyltransferase I-negative HEK293S stable mammalian cell line. Proc. Natl. Acad. Sci. U S A.

[b0055] Seiradake E., Harlos K., Sutton G., Aricescu A.R., Jones E.Y. (2010). An extracellular steric seeding mechanism for Eph-ephrin signaling platform assembly. Nat. Struct. Mol. Biol..

[b0060] Szymanski S.L., Huff K.W., Patel A.D., Murray J.R., Feasby J. (2008). Automated application of a novel high yield, high performance tissue culture flask. JALA.

[b0065] Tokunaga F., Hara K., Koide T. (2003). N-Linked oligosaccharide processing, but not association with calnexin/calreticulin is highly correlated with endoplasmic reticulum-associated degradation of antithrombin Glu313-deleted mutant. Arch. Biochem. Biophys..

[b0070] Walter T.S., Diprose J.M., Brown J., Pickford M.G., Owens R.J. (2003). A procedure for setting up high-throughput nano-litre crystallization experiments. I. Protocol design and validation. J. Appl. Crystalogr..

[b0075] Walter T.S., Diprose J.M., Mayo C.J., Siebold C., Pickford M.G. (2005). A procedure for setting up high-throughput nanolitre crystallization experiments. Crystallization workflow for initial screening, automated storage, imaging and optimization. Acta Crystallogr. Sect. D.

