# Supplementary material for: Automation of large scale transient protein expression in mammalian cells
Source: J Struct Biol. 2011 Aug;175(2-2):209–15. doi: 10.1016/j.jsb.2011.04.017 (PMC3477309; doi:10.1016/j.jsb.2011.04.017)

Supplementary Fig.1: Automated cell maintenance routine.

```
-<SelecT_Protocol>
- <properties>
  <description>New Protocol</description>
  </properties>
- <steps>
- <fetch maxrepeat="4">
  <dump pause="8s" />
  <dispense liquid="PBS" volume="15ml" />
  <swirl repeat="1" speed="100%" pause="0s"
capped="no" />
  <dump pause="10s" />
  <dispense liquid="trypsin" volume="5ml" />
  <swirl repeat="1" speed="100%" pause="0s"
capped="yes" />
  <incubate period="3min" />
  <shake repeat="10" speed="40%" pause="0s"
capped="yes" />
  <shake repeat="15" speed="100%" pause="0s"
capped="yes" />
  <dispense liquid="DMEM + 10%FBS"
volume="27ml" />
  <swirl repeat="1" speed="100%" pause="0s"
capped="no" />
  <putdown name="Master mix" />
- <new repeat="5" interleave="1" name="output">
  <putdown name="293T 1:5 split" />
  <mix volume="10ml" repeat="1"
fromheight="2mm" toheight="2mm"
mixspeed="5ml/s" finaldispensespeed="5ml/s"
pause="0s" newtip="no" name="Master mix" />
  <pipette volume="6ml" aspiratespeed="5ml/sec"
dispensespeed="5ml/sec" pause="0s" newtip="no"
fromname="Master mix" toname="293T 1:5 split"
fromheight="2mm" toheight="50mm" />
  <pickup name="293T 1:5 split" />
  <dispense liquid="DMEM + 10%FBS"
volume="24ml" />
  <swirl repeat="1" speed="100%" pause="0s"
capped="yes" />
  <store />
  </new>
  <pickup name="Master mix" />
  <dispose />
  </fetch>
  </steps>
  </SelecT_Protocol>
```

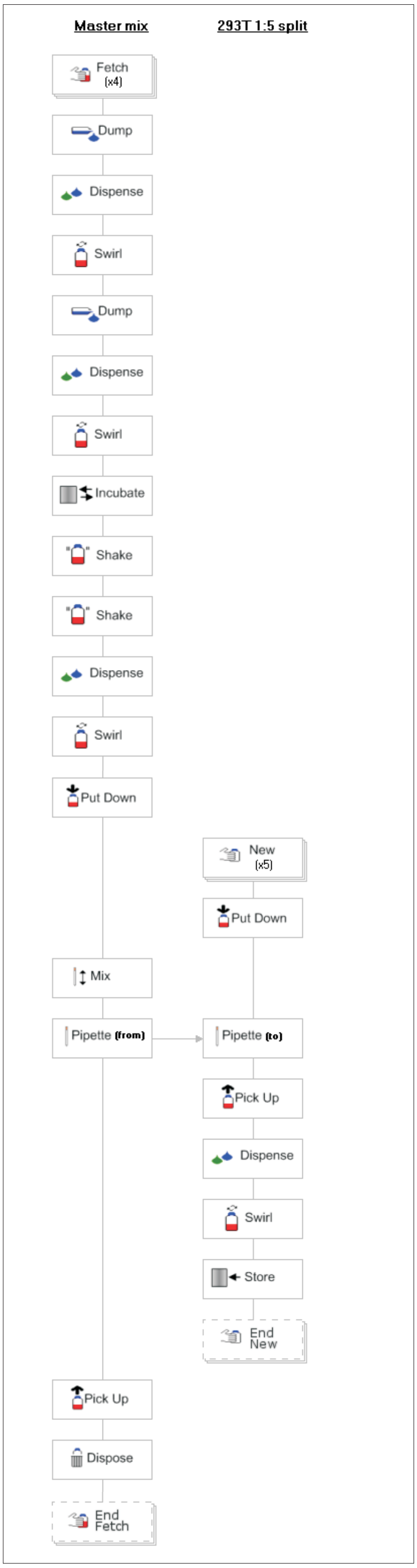

Supplementary Fig.2: Routine for automated seeding of triple flasks from a T175 flask.

```
- <SelecT_Protocol>
- <properties>
    <description>New Protocol</description>
    </properties>
- <steps>
- <fetch maxrepeat="1">
    <dump pause="7s" />
    <dispense liquid="PBS" volume="10ml" />
    <swirl repeat="1" speed="100%" pause="0s"
capped="no" />
    <dump pause="7s" />
    <dispense liquid="trypsin" volume="5ml" />
    <swirl repeat="1" speed="100%" pause="0s"
capped="yes" />
    <incubate period="3min" />
    <shake repeat="10" speed="60%" pause="0s"
capped="yes" />
    <shake repeat="15" speed="100%" pause="0s"
capped="yes" />
    <dispense liquid="DMEM + 10%FBS" volume="28ml"
/>
    <swirl repeat="1" speed="100%" pause="0s"
capped="no" />
    <putdown name="master" />
- <new repeat="3" interleave="1"
flasktypegroup="Triple">
    <putdown name="new triple" />
    <mix volume="10ml" repeat="2" fromheight="2mm"
toheight="2mm" mixspeed="5ml/s"
finaldispensespeed="5ml/s" pause="0s" newtip="no"
name="master" />
    <pipette volume="10ml" aspiratespeed="5ml/sec"
dispensespeed="5ml/sec" pause="0s" newtip="no"
fromname="master" toname="new triple"
fromheight="2mm" toheight="5mm" />
    <pickup name="new triple" />
    <dispense liquid="DMEM + 10%FBS"
volume="100ml" />
    <flip repeat="1" speed="100%" pause="0s"
capped="yes" />
    <swirl repeat="1" speed="100%" pause="0s"
capped="yes" />
    <flip repeat="1" speed="100%" pause="0s"
capped="yes" />
    <store />
    </new>
    <pickup name="master" />
    <dispose />
    </fetch>
    </steps>
    </SelecT_Protocol>
```

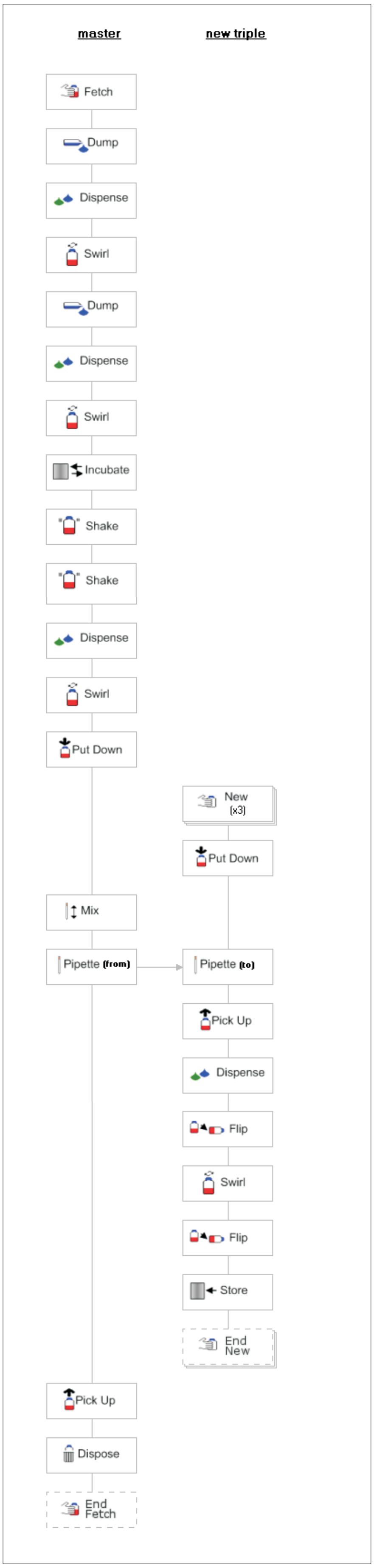

Supplementary Fig.3: Routine for automated seeding of a HYPERFlask from a T175 flask.

```xml
- <SelecT_Protocol>
- <properties>
    <description>New Protocol</description>
    </properties>
- <steps>
- <fetch maxrepeat="6">
    <dump pause="8s" />
    <dispense liquid="PBS" volume="15ml" />
    <swirl repeat="1" speed="100%" pause="0s" capped="no"
/>
    <dump pause="10s" />
    <dispense liquid="trypsin" volume="5ml" />
    <swirl repeat="1" speed="100%" pause="0s"
capped="yes" />
    <incubate period="3min" />
    <shake repeat="10" speed="50%" pause="0s"
capped="yes" />
    <shake repeat="15" speed="100%" pause="0s"
capped="yes" />
    <dispense liquid="DMEM + 10%FBS" volume="37ml" />
    <swirl repeat="1" speed="100%" pause="0s" capped="no"
/>
    <putdown name="mastermix" />
- <new repeat="1" interleave="1" flasktypegroup="Multiple">
    <putdown name="hyper" />
    <pipette volume="40ml" toheight="5mm"
aspiratespeed="5ml/sec" dispensespeed="3ml/sec"
pause="0s" newtip="no" fromname="mastermix"
toname="hyper" fromheight="2mm" />
    <pickup name="hyper" />
    <equilibrate repeat="1" speed="80%" pause="1s" />
    <dispense liquid="DMEM + 10%FBS" volume="505ml"
robotspeed="50%" />
    <dispense liquid="DMEM + 10%FBS" volume="10ml"
robotspeed="40%" />
    <hyperswirl repeat="1" speed="30%" pause="1s" />
    <store />
    <pickup name="mastermix" />
    <dispose />
    </new>
    </fetch>
    </steps>
    </SelecT_Protocol>
```

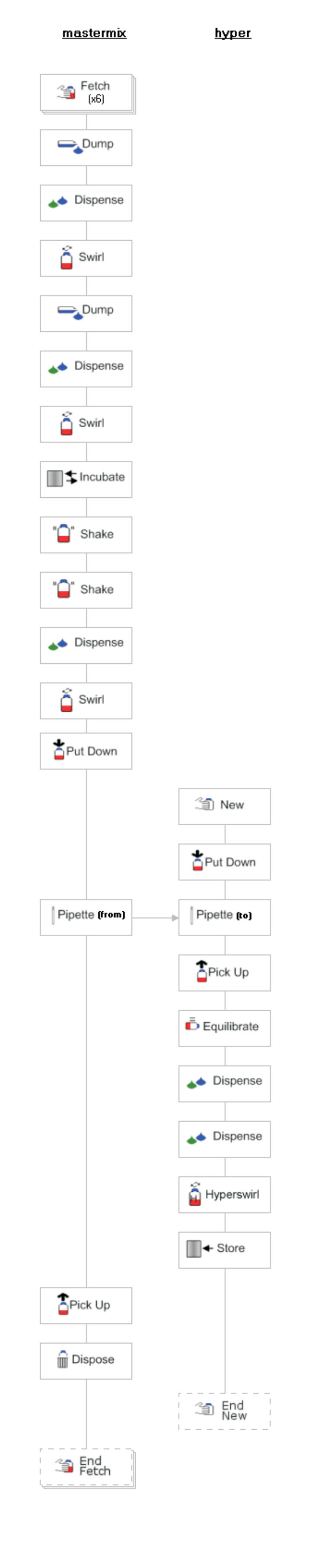

Supplementary Fig.4: Routine for automated transient transfection in a Triple flask.


```
- <SelecT_Protocol>
- <properties>
   <description>New Protocol</description>
   </properties>
- <steps>
- <fetch maxrepeat="12">
   <dump pause="5s" />
   <dispense liquid="PEI-DNA-DMEM-transfection-
Mixture" volume="30ml" />
   <flip repeat="1" speed="100%" pause="0s"
capped="yes" />
   <store />
   </fetch>
   </steps>
   </SelecT_Protocol>
```

```
- <SelecT_Protocol>
- <properties>
   <description>New Protocol</description>
   </properties>
- <steps>
- <fetch maxrepeat="12">
   <dispense liquid="DMEM + 10%FBS" volume="16ml"
/>
   <dispense liquid="dmem" volume="54ml" />
   <swirl repeat="1" speed="100%" pause="0s"
capped="yes" />
   <flip repeat="1" speed="100%" pause="0s"
capped="yes" />
   <store />
   </fetch>
   </steps>
   </SelecT_Protocol>
```


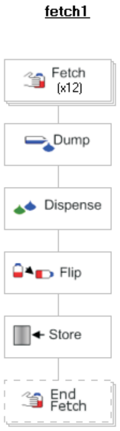

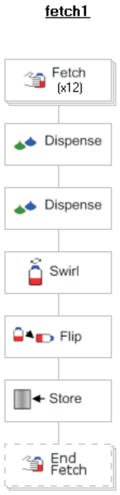

Supplementary Fig.5: Routine for automated HYPERFlask transfection.


```
- <SelecT_Protocol>
- <properties>
  <description>New Protocol</description>
  </properties>
- <steps>
- <fetch maxrepeat="1">
  <dump pause="7s" />
  <dispense liquid="PEI-DNA-DMEM-transfection-
Mixture" volume="100" />
  <equilibrate repeat="1" speed="100%"
pause="1s" />
  <dispense liquid="dmem" volume="344" />
  <dispense liquid="DMEM + 10%FBS"
volume="100ml" robotspeed="50%" />
  <dispense liquid="DMEM + 10%FBS"
volume="11ml" robotspeed="50%" />
  <hyperswirl repeat="1" speed="30%" pause="1s"
/>
  <store passage="no" />
  </fetch>
  </steps>
```


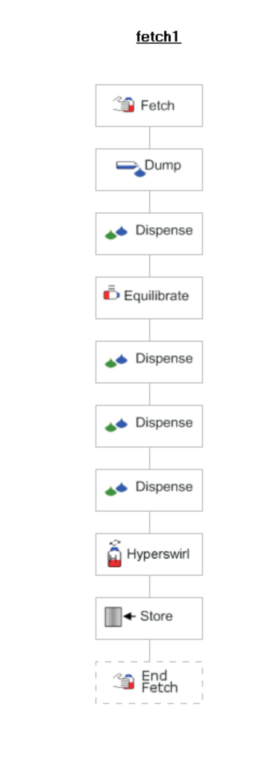

Supplement: Supplementary data 1 [file mmc1.pdf]
